# Intramammary infections with *Corynebacterium* spp. in bovine lactating udder quarters

**DOI:** 10.1371/journal.pone.0270867

**Published:** 2022-07-07

**Authors:** Anneke Lücken, Svenja Woudstra, Nicole Wente, Yanchao Zhang, Volker Krömker

**Affiliations:** 1 Faculty of Mechanical and Bioprocess Engineering, Department of Microbiology, University of Applied Sciences and Arts, Hannover, Germany; 2 Department of Veterinary and Animal Sciences, University of Copenhagen, Copenhagen, Denmark; University of Lincoln - Brayford Campus: University of Lincoln, UNITED KINGDOM

## Abstract

*Corynebacterium* spp. are frequently detected in bovine quarter milk samples, yet their impact on udder health has not been determined completely. In this longitudinal study, we collected quarter milk samples from a dairy herd of approximately 200 cows, ten times at 14 d intervals. Bacteriologically, Catalase-positive and Gram-positive rods were detected in 22.7% of the samples. For further species diagnosis, colonies were analyzed by MALDI-TOF MS. *Corynebacterium bovis*, *C*. *amycolatum*, *C*. *xerosis* and 10 other *Corynebacterium* spp. were detected. The three aforementioned species accounted for 88.4%, 8.65% and 0.94% of all cultured *Corynebacterium* spp., respectively. For further evaluation of infection dynamics, the following three infection definitions were applied: A (2/3 consecutive samples positive for the same species), B (≥1000 cfu/mL in one sample), C (isolated from a clinical mastitis case). Infections according to definition B occurred most frequently and clinical mastitis with *Corynebacterium* spp. occurred once during sampling. Life tables were used to determine the duration of infection. According to infection definition A, infection durations of 111 d and 98 d were obtained for *C*. *bovis* and *C*. *amycolatum*, respectively. Exemplarily, longer lasting infections were examined for their strain diversity by RAPD PCR. A low strain diversity was found in the individual quarters that indicates a longer colonization of the udder parenchyma by *C*. *bovis* and *C*. *amycolatum*.

## Introduction

Despite ever-increasing efforts to prevent bovine mastitis, it remains one of the most important diseases on dairy farms. Besides individual and environmental factors, pathogens play a role in the cause of mastitis [1]. In addition to clinical cases, subclinical mastitis also plays an important economic role, as it results in increased somatic cell count and decreased milk yield [2].

*Corynebacterium* spp. are frequently detected in milk samples, but it usually remains unclear whether this is due to infection, non-pathogenic colonization or contamination. While colonization of the teat canal alone is more indicative of contamination [3], *Corynebacterium* spp. have also been detected in subclinical mastitis with significantly elevated cell counts and in cases of clinical mastitis [4–6]. On the other hand, *Corynebacterium* spp. are also regularly isolated from milk samples of cows without any indication of mastitis [7]. Species diagnostics are performed only rarely for mastitis pathogens. A previous study of ours already showed that, in addition to *C*. *bovis*, *C*. *amycolatum* also accounts for a relevant proportion of *Corynebacterium* spp. detections, and also other species can be isolated from bovine milk samples [5]. So far, there have been hardly any insights into long-term infections caused by this genus. *Corynebacterium* spp. positive findings are sometimes only perceived as incidental findings and are not interpreted further. In part, they are even said to have a protective effect with a protective effect against major pathogens [8]. In contrast, other authors describe that the timing of infection with *Corynebacterium* spp. has an impact on whether the infection has a protective or even opposing effect [9].

It is apparent that the exact role of *Corynebacterium* spp. in relation to udder health is not yet clear. In order to assess whether an intramammary infection (IMI) is present, a definition of IMI due to *Corynebacterium* spp‥ must first be established. Rachah *et al*. [10] had previously attempted to examine transmission dynamics of *Corynebacterium* spp. using different infection definitions and found that mid-lactation was associated with the highest rate of new infection, but they did not distinguish between different species. Older literature in particular mentions dry cow therapy and post milking teat disinfection as herd prevalence reducing agents, indicating that *Corynebacterium* spp. belong to the cow-associated pathogens (e.g., [11]). Knowledge of the duration of infection at species level is important for further understanding of infections. It can give an indication of self-cure and thus be crucial for defining treatment strategies. Taking antibiotic reduction into account, it must be assessed whether antibiotic treatment is useful and necessary. To obtain this information, a longitudinal study is necessary to gain more insight into the dynamics of infections.

In addition to microbiological methods, molecular biological methods are also important to understand the epidemiology of IMI with *Corynebacterium* spp. [12].

The aim of the present study was to describe the infection dynamics of *Corynebacterium* spp. observed in a longitudinal study in one dairy cow herd using modern methods such as matrix-assisted laser detection ionization time of flight mass spectrometry (MALDI-TOF MS) for species diagnostics and randomly amplified polymorphic DNA polymerase chain reaction (RAPD PCR) for strain-level comparison.

## Materials and methods

### Milk sampling and examination

For this longitudinal study, we chose a dairy farm in Sweden with about 200 lactating Holstein Friesian, Swedish Red or crossbreed cows. The cows were kept in a free stall barn with high cubicles and were milked twice daily in a herringbone milking parlor with post milking teat disinfection. Drying off was done gradually over seven days, with individual quarter selective drying off. In the present study, all applicable guidelines for the care and use of animals were considered. Approval was granted by the animal welfare committee of the university (University of Veterinary Medicine Hannover, Foundation; file reference: TVO-2020-V-60) on 3rd June 2020. According to the local government, an application for animal testing was not necessary. The International Guiding Principles for Biomedical Research Involving Animals (1985) were followed. The farm was sampled from June to October 2020. Quarter foremilk samples were taken aseptically from the entire herd ten times at 14-day intervals. They were then cooled and taken to the laboratory in Hannover, Germany, where they were prepared the following day. A total of 10μL of each milk sample was spread on one quarter of an esculin blood agar (Oxoid Deutschland GmbH, Wesel, Germany) and incubated at 37°C. The milk samples were examined microbiologically after 24h and 48h in accordance with the Guidelines of the German Veterinarian Association [13]. Samples were classified as contaminated if more than two different colonies per quadrant of the plate could be identified. Catalase-positive and Gram-positive rods were preselected as this corresponds to the characteristics of corynebacteria in order to keep the number of isolates for species diagnostics within limits. Nevertheless, other bacteria can also be isolated according to these criteria. The number of colony-forming units (cfu) was determined semiquantitatively at the initial smear. From samples with the previously described characteristics, one colony each was re-smeared on blood agar and regrown. Species identification was performed by BioTyper™ matrix-assisted laser desorption/ionization time-of-flight mass spectrometry (MALDI-TOF MS; Bruker Daltonik GmbH, Bremen, Germany; Microflex LT/SH smart). The identification was carried out after direct transfer without formic acid extraction, using the MBT Compass Library (Revision F, MBT 8468 MSP Library). The cut-off for species level was reduced from 2.0 to ≥1.7 in accordance with Theel *et al*. [14]. Bacteria other than *Corynebacterium* spp. were not considered for further evaluation. Somatic cell count was determined by flow cytometry (SomaScope Smart®, Delta Instruments B.V., Drachten, the Netherlands). Isolates were preserved at -80°C with 800 μL of brain heart broth (Merck KGaA, Darmstadt, Germany) and 200 μL of glycerol. The amount of milk was recorded during the first four weeks of the sampling period, and based on this, the average amount of milk per cow per day was determined.

### Infection definitions

Three different infection definitions were applied. These were based on the study by Rachah *et al*. [10]. According to infection definition A, an intramammary infection (IMI) was assumed if the same species could be isolated with at least 500 cfu/mL in two of three consecutive samples. IMI by definition B was present if at least 1000 cfu/mL of *Corynebacterium* spp. grew on the agar. The third infection definition referred to the clinical status of the cow and was applicable when one quarter had a finding of *Corynebacterium* spp. in clinical mastitis regardless of the amount of colony-forming units per milliliter.

### Infection duration

Infection durations were determined separately for each infection definition and species. Due to the bi-weekly sampling rhythm, an onetime isolation of *Corynebacterium* spp. was recorded with an infection duration of 14 days, since this corresponds to the time interval between the midpoints of two sampling dates [15]. Accordingly, only values divisible by 14 were present IMI were treated as new infections if either no *Corynebacterium* spp. or a different *Corynebacterium* spp. was detected on at least two consecutive samplings (definition A) or if this was the case on one sampling (definition B). According to definition A, one negative sample between two positive samples did not interrupt the infection. Since the sampling lasted only for part of the lactation, the data were partially censored on the right and/or left. First, microbiological criteria were used to determine the individual infection durations and then the median duration was determined.

### Teat tip skin swabs

In addition to milk samples, a few skin swabs were taken from the teat tip of cows with *Corynebacterium* spp. detection at an earlier sampling. After dry pre-cleaning of the teat tip skin had been performed, a dry sterile swab was rotated around the teat canal opening before milking. The swab was then kept in sterile Ringer’s solution. Ringer’s solution of each swab was diluted at levels 10^−1^,10^−2^ and 10^−3^ and 100 μL thereof was inoculated on esculin blood agar (Oxoid Deutschland GmbH) and then incubated at 37°C for 48 h. All plates were examined as previously described for the milk samples, and Catalase-positive and Gram-positive rods were grown again on blood agar to obtain pure cultures. These isolates were further examined and the species were determined by MALDI-TOF MS as with the milk samples. The isolates were preserved like those of the milk samples for further investigation.

### Strain comparisons with Random Amplified Polymorphic DNA Polymerase Chain Reaction

Exemplary different isolates of the three species *C*. *bovis*, *C*. *amycolatum* and *C*. *testudinoris* were analyzed for strain diversity comparative at quarter level and cow level, among different cows, and in comparison to isolates from teat tip skin by Random Amplified Polymorphic DNA Polymerase Chain Reaction (RAPD PCR). For this purpose, 10 μL of the preserved broth from each isolate was spread on one quarter of a blood agar plate and incubated at 37°C for 72h. DNA was extracted using the DNeasy Blood and Tissue Kit (Qiagen N.V., Venlo, the Netherlands) following the manufacturer’s instructions. For the species *C*. *bovis*, the primer ERIC-1R (5’-ATGTAAGCTCCTGGGGATTCAC-3’) [16] was used, whereas for *C*. *amycolatum* and *C*. *testudinoris*, the M13 forward primer (5’-GTA AAA CGA CGG CCAGT-3’) [17] was utilized. The temperature profile for ERIC-1R was modified to 35 cycles of denaturation for 1 min at 94°C, primer annealing for 1 min at 25°C, elongation for 4 min at 72°C, and a final extension for 8 min at 72°C. RAPD PCR was performed using a 25 μL reaction volume. This contained 12.5 μL ReadyMix™ Taq PCR Reaction Mix (Sigma-Aldrich Chemie GmbH, Taufkirchen, Germany), 20 pmol of primer as mentioned, 5 μL of DNA extract, and replenished to 25 μL with water. RAPD PCR was performed in the Mx3005P qPCR system (Agilent Technologies Inc., Santa Clara, CA, USA). After RAPD PCR, 2 μL of MIDORI^Green^ Direct (NIPPON Genetics Europe GmbH, Düren, Germany) was added to each reaction batch for staining. The product was then pipetted individually into a 2% agarose gel and gel electrophoresis was performed for 2 h at 150 V. Identical RAPD patterns of PCR products were defined as the same strain. Several gel electrophoreses were performed. First, individual cows and quarters were considered, and then the respective strains of several cows and quarters were comparatively examined in another gel.

### 16S rRNA gene amplification and sequencing

To verify the MALDI-TOF MS species identification results, a portion of the isolates were analyzed for species by 16s rRNA sequencing in another laboratory (Section for Veterinary Clinical Microbiology, Copenhagen University) Here, the DNeasy Blood and Tissue Kit (Qiagen N.V., Venlo, the Netherlands) was also used and DNA extracted according to [18]. The full-length 16S rRNA was amplified using DreamTaq Green PCR Master Mix (ThermoFisher Scientific) with primers 8F (AGAGTTTGATCCTGGCTCAG) and 1492R (GGTTACCTTGTTACGACTT). The PCR was carried out in the ProFlex PCR System (Applied Biosystems) under the following conditions: 2 min at 95°C; 30 cycles of 30 s at 95°C, 30 s at 52°C, and 90 s at 72°C; 5 min at 72°C. The genome of Escherichia coli ATCC 25922 was included as positive control. The PCR products were purified using the GeneJET PCR Purification Kit (ThermoFisher Scientific) according to the manufacturer’s instructions. The PCR products were visualised on a 1% agarose gel containing ethidium bromide and GeneRuler 1 kb Plus DNA Ladder (ThermoFisher Scientific) to ensure fragment lengths approximating 1484 bp. DNA concentration of the sequence was determined by the Qubit quantification system (Life Technologies). PCR products were double-strand sequenced, and sequences were blasted against the NCBI nr database.

### Statistical analysis

The collection and processing of data were carried out with Microsoft excel (Microsoft Corp., 2010). For analyzing the dataset, the program SPSS 26.0 (IBM Inc., Armonk, NY, USA) was used with quarter milk samples being considered as the statistical unit. Life tables were used to determine the duration of infection, taking into account the right- and left-censored data. To determine a dependence between the lactation number and the duration of infection of each species, a Wilcoxon test was performed.

## Results

In the entire sampling period, 263 different cows were sampled. The average amount of milk per cow per day was 33.5 L. Due to the lack of data on milk solids, this value was not corrected for energy. The geometric mean of the measured somatic cell counts was between 25,000 SCC/mL and 39,800 SCC/mL at the individual sampling points. *Corynebacterium* spp. were detected in at least one quarter of 212 cows (80.6%). Of a total of 1,052 sampled quarters, 524 (49,8%) had at least one coryneform positive finding. Accordingly, an average of more than two quarters per cow were affected (2.47 quarters/cow). In 22.7% of all samples, Catalase-positive and Gram-positive rods could be isolated. Further species diagnostics by MALDI-TOF MS detected species other than Corynebacterium in 0.03% (n = 47), these were excluded in further investigation. Using MALDI-TOF MS, for 1,595 of the 1,764 isolates, the species of *Corynebacterium* spp. could be determined. The remaining 169 isolates could not be clearly identified by MALDI-TOF MS. A total of 13 different *Corynebacterium* spp. were detected (Table 1). With almost 90%, *C*. *bovis* made up the majority of *Corynebacterium* spp. isolates. *C*. *amycolatum* was the second most common with 8.65%, while *C*. *xerosis*, the third most common species, accounted for just under 1%. The remaining ten species together accounted for approximately 2% of all the isolates.

**Table 1:** Distribution of *Corynebacterium* spp. isolated from bovine quarter milk samples.

	Number of isolates	Percentage of all *Corynebacterium* spp. isolates
***Corynebacterium amycolatum***	138	8.65
***Corynebacterium bovis***	1410	88.40
***Corynebacterium confusum***	2	0.13
***Corynebacterium frankenforstense***	13	0.81
***Corynebacterium freneyi***	1	0.06
***Corynebacterium jeikeium***	1	0.06
***Corynebacterium kroppenstedtii***	2	0.13
***Corynebacterium pilosum***	1	0.06
***Corynebacterium stationis***	3	0.19
***Corynebacterium suicordis***	2	0.13
***Corynebacterium testudinoris***	6	0.38
***Corynebacterium variabile***	1	0.06
***Corynebacterium xerosis***	15	0.94
***total***	**1595**	**100**

### Number of infections

Table 2 shows the number of infections per species subdivided according to the three infection definitions. Infection definition A, at least two positive findings of the respective species in three consecutive samplings, could be applied mainly to *C*. *bovis* and *C*. *amycolatum*. A total of 24 and 347 infections according to this infection definition occurred with *C*. *amycolatum* and *C*. *bovis*, respectively. The number of infections for these two species according to definition B, at least 1000 cfu/mL in one sample, was approximately twice as high. In addition, this definition could also be applied to the eleven other isolated species. Clinical mastitis and consequently definition C occurred only once in *C*. *bovis*. In some cases with the species *C*. *amycolatum*, *C*. *bovis*, *C*. *frankenforstense*, *C*. *stationis* and *C*. *xerosis*, bacteriological evidence occurred without any of the three definitions of infection applying.

**Table 2:** Number of observed *Corynebacterium spp*. IMIs according to the infection definitions A, B and C.

	Definition A: two of three *Corynebacterium* spp. positive	Definition B: ≥ 1000 cfu/mL	Definition C: clinical mastitis	No definition applicable
	- number of infections -			
*Corynebacterium amycolatum*	24	69	0	13
*Corynebacterium bovis*	347	800	1	26
*Corynebacterium confusum*	0	2	0	0
*Corynebacterium frankenforstense*	0	6	0	6
*Corynebacterium freneyi*	0	1	0	0
*Corynebacterium jeikeium*	0	1	0	0
*Corynebacterium kroppenstedtii*	1	2	0	0
*Corynebacterium pilosum*	0	1	0	0
*Corynebacterium stationis*	0	2	0	1
*Corynebacterium suicordis*	0	2	0	0
*Corynebacterium testudinoris*	0	6	0	0
*Corynebacterium variabile*	0	1	0	0
*Corynebacterium xerosis*	0	14	0	1

In this table, the total number of infections occurring is shown, ordered by the respective infection definition and species.

### Infection duration

According to the study protocol, the infection durations followed a 14-day rhythm from 14 to 140 days. The data were partially censored on the right and/or left. Table 3 shows the infection durations of *C*. *bovis*, *C*. *amycolatum*, and *C*. *xerosis*. In each case, the complete data set and the uncensored data set are considered. The infection definition A could be applied mainly to *C*. *bovis* and *C*. *amycolatum*. The minimum infection period for definition A was 28 days. *C*. *bovis* could be detected in 16 quarters over the entire sampling period, corresponding to an infection duration of at least 140 days. The median for *C*. *bovis* was 56 days for infection definition A for the complete data. For *C*. *amycolatum*, the maximum infection duration was 98 days, and the median for definition A was 42 days.

**Table 3:** Frequency of infections and infection durations of *Corynebacterium bovis*, *Corynebacterium amycolatum* and *Corynebacterium xerosis*.

Species, infection definition	Number of infections	14	28	42	56	70	84	98	112	126	140	Median
***Corynebacterium bovis***												
**Definition A—all**	347	-	96	63	45	46	25	24	19	13	16	56
**Definition A–excl. censored**	123	-	43	27	17	17	10	4	5	0	0	42
**Definition B—all**	800	474	168	82	37	22	8	4	1	2	2	14
**Definition B–excl. censored**	463	299	90	40	19	12	2	1	0	0	0	14
**Definition C**	1	1	0	0	0	0	0	0	0	0	0	14
***Corynebacterium amycolatum***												
**Definition A–all**	24	-	9	5	1	2	4	3	0	0	0	42
**Definition A–excl. censored**	10	-	3	4	0	0	1	2	0	0	0	42
**Definition B—all**	69	44	16	3	4	0	2	0	0	0	0	14
**Definition B–excl. censored**	39	26	8	3	1	0	1	0	0	0	0	14
***Corynebacterium xerosis***												
**Definition B—all**	14	14	0	0	0	0	0	0	0	0	0	14
**Definition B–excl. censored**	9	9	0	0	0	0	0	0	0	0	0	14

Infection definition B allowed for short infections, so the minimum infection duration was 14 days. While the maximum duration of infection after this infection definition was 140 days for *C*. *bovis*, *C*. *amycolatum* reached a maximum duration of infection of 84 days and *C*. *xerosis* of 14 days under this infection definition. Overall, primarily single detections occurred under infection definition B, resulting in a median of 14 days for all three species mentioned under this definition.

Life Tables were created to put the censoring of infection durations into some perspective. These Life Tables were used to determine the median survival time for each species and infection definition as listed in Table 4.

**Table 4:** Median survival time (Life Tables) of the different *Corynebacterium* spp. infections.

	Definition A: two of three *Corynebacterium* spp. positive	Definition B: ≥ 1000 cfu/mL
	- median survival time (days) -	
*Corynebacterium amycolatum*	98	32
*Corynebacterium bovis*	111	34
*Corynebacterium confusum*	-	21
*Corynebacterium frankenforstense*	-	25
*Corynebacterium freneyi*	-	21
*Corynebacterium jeikeium*	-	21
*Corynebacterium kroppenstedtii*	49	21
*Corynebacterium pilosum*	-	21
*Corynebacterium stationis*	-	14
*Corynebacterium suicordis*	-	21
*Corynebacterium testudinoris*	-	14
*Corynebacterium variabile*	-	14
*Corynebacterium xerosis*	-	23

The analysis with Wilcoxon tests showed a dependency of infection duration for the species *C*. *bovis* and *C*. *amycolatum* on the lactation number for infection definition B. Under the described conditions, the infection durations in the second lactation were significantly longer than in the first lactation (p<0.5). For infection definition A, neither species nor lactation number had a significant effect on infection duration.

### Random amplified polymorphic DNA polymerase chain reaction

#### Corynebacterium bovis

As an example, 73 isolates from 14 quarters with prolonged infections with *C*. *bovis* were examined for strain diversity. These belonged to a total of eight cows, of which all four quarters of two cows were also comparatively examined. For example, when comparing multiple isolates from one cow, we were able to identify only two different strains based on the banding pattern. While one strain (5.b.II) occurred at several time points and in all four quarters of this cow, the second strain (6.b.) occurred only in one sampling in one quarter. A total of eight different strains were detected among the 73 isolates, but only a maximum of two different strains in one quarter. 5.b.I, 5.b.II and 1.b. occurred most frequently, the remaining strains only sporadically.

#### Corynebacterium amycolatum

**Fig 1:**
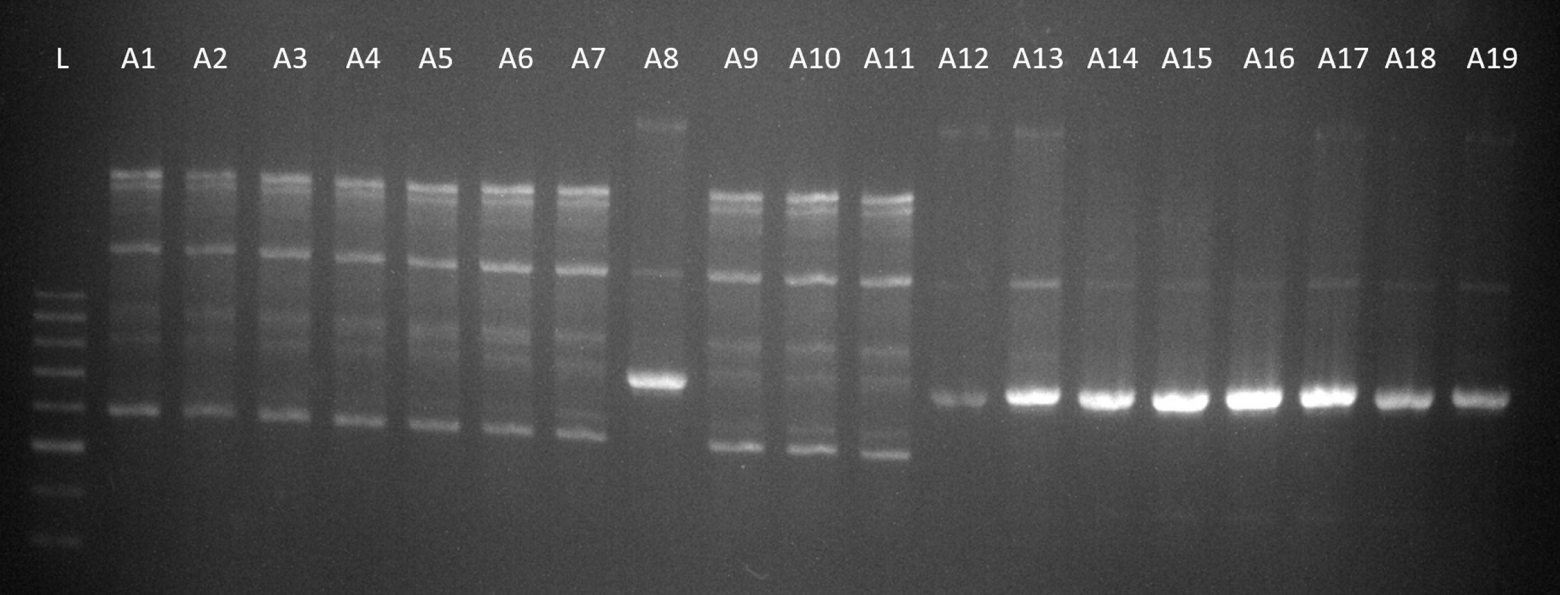
*Corynebacterium amycolatum* RAPD PCR types. L = ladder, strain 1.a = A1-A7, A9-A11; 2.a = A8, A12-A19^**a**^. ^a^A7, A8 are isolates from teat skin swabs.

Strain diversity was considered in prolonged infections of *C*. *amycolatum* in six quarters of five cows. A total of two different strains were detected in the milk samples, but only one strain per quarter. The two strains were also found in the teat skin swabs of one quarter, and one of these strains was detected in its milk samples during six samplings (Fig 1).

#### Corynebacterium testudinoris

*C*. *testudinoris* could be determined in three different quarters from one cow at the same sampling day. All three isolates showed a similar banding pattern in RAPD PCR after amplification with the M 13 primer.

### 16s rRNA gene amplification and sequencing

Before forwarding the isolates for sequencing, species differentiation was again performed by MALDI-TOF MS. The same species was repeatedly detected and also confirmed by 16S rRNA sequencing (Table 5).

**Table 5:** Results of 16S rRNA sequencing.

Strain	Species identified by MALDI-TOF MS	Score value MALDI-TOF	Query cover (%) forward/reverse	Identity (%) forward/reverse	Sequence length (n) forward/reverse
***Corynebacterium bovis***					
**1b**	*Corynebacterium bovis*	1,78	85/85	97/97	1495/1527
**2b**	*Corynebacterium bovis*	1,94	83/85	98/97	1482/1522
**3b**	*Corynebacterium bovis*	1,86	82/85	96/97	1641/1496
**4b**	*Corynebacterium bovis*	2,14	86/94	97/97	1487/1218
**5b1**	*Corynebacterium bovis*	1,99	76/83	98/96	1471/1513
**5b2**	*Corynebacterium bovis*	2,05	78/91	97/97	1567/1400
**6b**	*Corynebacterium bovis*	2,04	83/82	96/98	1554/1501
***Corynebacterium amycolatum***					
**1a**	*Corynebacterium amycolatum*	2,32	74/91	91/86	1238/1283
**2a**	*Corynebacterium amycolatum*	2,20	66/81	82/97	913/1513

## Discussion

To evaluate the infection dynamics of *Corynebacterium* spp., we sampled all lactating cows on one farm ten times over an 18-week-period. In addition to routine diagnostics, we also determined the species of each *Corynebacterium* spp. isolate using matrix assisted laser detection ionization time of flight mass spectrometry (MALDI-TOF MS). As previously noted by our research group, *C*. *amycolatum* is the second most present species in addition to *C*. *bovis*, but other species can also be detected [5]. In the present study, an even higher species diversity could be detected, although only one farm was investigated. This finding further reinforces the importance of conducting species-specific testing.

### Infection duration

Infections occurred more than twice as often when applying definition B than under definition A. For definition B, a single detection of 1000 cfu/mL was sufficient. Single detections occurred most frequently and corresponded to an infection duration of 14 days. Regardless of censoring, the frequent occurrence of such short infections resulted in a median infection duration by definition B of 14 days. Since almost only *C*. *bovis* and *C*. *amycolatum* occurred more than one time in a quarter, which was necessary for an infection according to definition A (2/3 consecutive samples positive for the same species), infections according to this definition were only observed for these species, except for one case in *C*. *kroppenstedtii*. Likewise, it cannot be ruled out that the test methods may detect some species less effectively or not at all, with the result that fewer and shorter infections with these species occurred accordingly. Since a single detection of the microorganism was not sufficient under definition A, the minimum infection period for definition A was 28 days. According to definition A, a negative sample between two *Corynebacterium* spp. positive samplings did not interrupt an infection, so that altogether longer infections were possible. According to definition B, a negative sample between two *Corynebacterium* spp. positive samplings resulted in two shorter infections. This is also reflected in the markedly longer median infection duration. Due to the study design, multiple censoring of infection durations was possible. The infections could have started before the first sampling as well as have lasted beyond the last sampling of individual animals (e.g., due to the study timeframe or the cow being in the dry period). Accordingly, right and left censoring occurred and infection durations longer than the observation period could not be determined correctly. To get a little closer to the real infection duration, life tables were created and the median survival time was generated. For example, the median survival time for infections according to definition A was 111 d and 98 d for *C*. *bovis* and *C*. *amycolatum*, respectively. With respect to infection definition B, the duration of infection defined as the median survival time was 35 d and 32 d for *C*. *bovis* and *C*. *amycolatum*, respectively. It is nonetheless possible that infection durations remained underestimated because sampling did not occur over a sufficiently long period of time. Furthermore, infections could be interrupted by contaminated samples or mixed infection samples, which were not included in this study as *Corynebacterium* spp. positive findings. Detection of *C*. *bovis* in clinical mastitis (definition C) occurred only once and thus also corresponded to an infection duration of 14 days. Likewise, it cannot be ruled out that the test methods may detect some species less effectively or not at all, with the result that fewer and shorter infections with these species occurred accordingly.

To date, to our knowledge, the duration of infection of *Corynebacterium* spp. has not been determined on a species-specific basis. Langoni *et al*. [19] determined an average infection duration between 111d and 197 d for *Corynebacterium* spp‥ In this aforementioned study, the herd was sampled monthly and also only the animals in which *Corynebacterium* spp. were detected during the first sampling were further sampled. Although the study design is not very comparable, they nevertheless appear to be consistent with the statement that *Corynebacterium* spp. can cause longer lasting infections. As *C*. *bovis* and *C*. *amycolatum* appear to be able to cause prolonged infections with sometimes low pathogen shedding, infection definition A is most likely to apply. Accordingly, infections with these two species cannot always be ruled out on the basis of one negative sample collection, and even detections with low pathogen excretion below the limit of 1000 cfu/mL (infection definition B) do not rule out a long-lasting infection. *Corynebacterium* spp. other than the two previously mentioned ones could usually only be detected once and showed a short duration of infection, which contradicts a longer term colonization of these species in the udder parenchyma. Assuming a prolonged infection of the udder parenchyma associated with signs of mastitis, treatment may be appropriate.

We found a dependence of infection duration on lactation number for *C*. *bovis* and *C*. *amycolatum* under infection definition B, which was correlated positively. Previous studies have found a higher prevalence of *Corynebacterium* spp. in multiparous cows [7,20]. These two aforementioned findings are consistent insofar as a longer duration of infection in multiparous animals also allows for a higher probability of detection, and thus a higher prevalence.

### Strain diversity

Since we wanted to make a statement about the duration of the infections, we had to find out whether the same strains were involved in longer lasting infections. The strain diversity of the isolates was investigated using randomly amplified polymorphic DNA polymerase chain reaction (RAPD PCR). To our knowledge, strain comparisons of *Corynebacterium* spp. obtained from bovine milk samples have not yet been performed. Brennan *et al*. [17] previously used the M13 primer for *C*. *amycolatum* isolated from smear cheese. The primer provided our study with a banding pattern in RAPD PCR for *C*. *amycolatum* and *C*. *testudinoris*; examinations with this primer were less successful for *C*. *bovis*. Subsequently, we followed another study investigating *C*. *pseudotuberculosis* and used the primer ERIC 1 R for *C*. *bovis* [16]. Using the aforementioned primers for RAPD PCR and subsequent gel electrophoresis, we were able to screen the species for strain diversity.

Since we only looked at a few quarters for their strain diversity during prolonged infections, we can only give a trend based on the strain comparisons. The same *C*. *bovis* strain could be found in several quarters of one cow, as well as at several time points in one quarter. The same could be observed in other cows or quarters. However, more than one strain was found in this herd. Strains 5.b.I and 5.b.II were very similar in their banding pattern and were considered as one strain in the evaluation. Thus, they occurred in five of the eight cows examined for strain diversity in *C*. *bovis* infections.

Analyzing *C*. *amycolatum* isolates, we were able to identify two different strains, both of which were found in the milk of two cows each and in the skin swabs of one cow. It is interesting to note that both strains in the skin swabs originated from one cow, but only strain I.a could be detected in its milk, and this over six sampling dates. This could indicate that the pathogens isolated from the milk are bacteria that primarily live on the teat skin, but some of them manage to settle in the udder and cause a permanent infection there or it could also be a case of contamination.

To confirm that the isolates examined for strain diversity were indeed different strains of one species and that the different banding patterns were not due to incorrect species identification by MALDI-TOF MS, 16S rRNA sequencing was performed. As can be seen in Table 5, the previously determined species matched the results of 16S rRNA sequencing.

The strain comparisons indicate that prolonged infections with *C*. *bovis* and *C*. *amycolatum* indeed usually involved one strain. This finding makes incidental detection caused by contamination of the sample unlikely. Due to the method of milk sampling, teat canal colonization as described by Bexiga *et al*. [3] cannot be ruled out completely. However, *Corynebacterium* spp. has also been detected in the teat cistern, glandular cistern, and glandular tissue in previous studies [21], allowing for prolonged infection of udder tissue.

The fact that one strain could be repeatedly detected in different cows, and indeed on several quarters of a cow, for both *C*. *bovis* and *C*. *amycolatum*, indicates a certain contagiousness and a potential for the pathogen to spread [12]. Despite the post-milking teat disinfection performed on this farm, which Brooks *et al*. [11] cited for prevalence reduction, *Corynebacterium* spp. was detected in 22.7% of the samples. This could be due to the dipping agent used. Possibly, other methods are still needed to reduce the prevalence of *Corynebacterium* spp‥

## Conclusion

Corynebacteria of the species *C*. *bovis* and *C*. *amycolatum* can lead to longer lasting infections of the udder parenchyma. If this is accompanied by symptoms of inflammation, treatment may be useful.

## Supporting information

S1 Table *Corynebacterium* spp. data set. In this table all infections are listed with their durations, definition and the respective species.
(XLSX)

S2 Table Results 16s rRNA gene amplification and sequencing. This table contains the 16s rRNA gene amplification and sequencing results presented in Table 5 of the manuscript.
(XLSX)

S1 File Ram image *Corynebacterium amycolatum* RAPD PCR types. This document contains the raw image from the comparison of the Corynebacterium amycolatum strains including the labeling (Fig 1).
(PDF)
