# Supplementary material for: Intramammary infections with Corynebacterium spp. in bovine lactating udder quarters
Source: PLoS One. 2022 Jul 7;17(7):e0270867. doi: 10.1371/journal.pone.0270867 (PMC9262192; doi:10.1371/journal.pone.0270867)

Original gel images - Intramammary infections with *Corynebacterium* spp. in bovine lactating udder quarters

1) Raw image Figure 1, *C. amycolatum* isolates from different cows

   RAPD PCR with M 13

L	A1	A2	A3	A4	A5	A6	A7	A8	A9	A10	A11	A12	A13	A14	A15	A16	A17	A18	A19

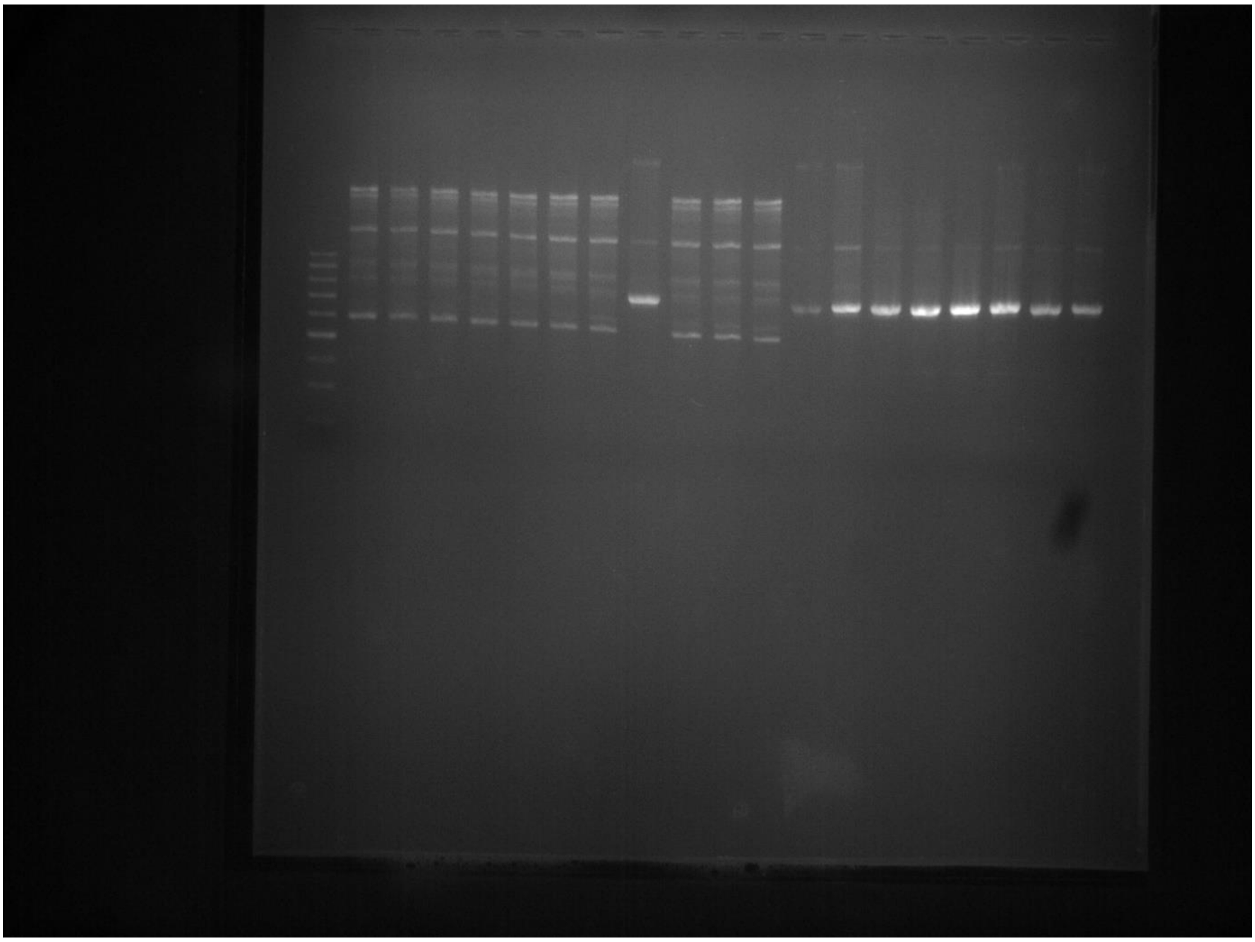

Supplement: S1 File — This document contains the raw image from the comparison of the Corynebacterium amycolatum strains including the labeling (Fig 1). (PDF) [file pone.0270867.s003.pdf]
